# A solitary hypothalamic metastasis from prostatic cancer mimicking a giant thrombotic aneurysm and presenting with intraventricular hemorrhage and acute hydrocephalus: a case report

**DOI:** 10.1186/s43055-020-00367-z

**Published:** 2020-12-11

**Authors:** Zarhra Saadatpour, Ali Rezaei, Aparna Singhal, Houman Sotoudeh, Kamran Tavakol

**Affiliations:** 1 Department of Neuroradiology, University of Alabama at Birmingham (UAB), 619, 19th Street S, Birmingham, AL 35294 USA; 2 College of Medicine, Howard University, Washington, DC, 20059 USA; 3 Marion, USA

**Keywords:** Prostatic neoplasm, Cerebral intraventricular hemorrhage, Neoplastic metastasis, Aneurysm, Hydrocephalus

## Abstract

**Background:**

Despite the high prevalence of prostate cancer, its brain parenchymal metastasis is not common and intracranial hemorrhage due to such a metastasis is even less common. This report presents a challenging case of solitary brain metastasis secondary to prostate cancer that gave rise to intraventricular hemorrhage and acute hydrocephalus mimicking a giant aneurysm.

**Case presentation:**

A 77-year-old man with a history of prostate cancer, hypertension, and morbid obesity presented to the emergency room with a severe headache. He was afebrile with a blood pressure of 144/79 mmHg, alert, without any sign of sensory or motor deficit. Shortly after admission, he became unresponsive and was immediately intubated. His blood tests revealed hypernatremia at 154 mmol/L; otherwise, the lab data including the COVID-19 screening proved normal. The cerebral CT and MR images, with and without contrast, were interpreted as a giant thrombotic aneurysm extending to the suprasellar region by the emergency radiologist. Also, moderate intraventricular hemorrhage, acute hydrocephalus, and sub-ependymal interstitial edema were observed. Upon further evaluation of the images, the lesion was determined to be an exophytic hemorrhagic hypothalamic mass, and the subsequent biopsy was consistent with prostate cancer metastasis.

**Conclusions:**

The exophytic hemorrhagic hypothalamic metastasis can mimic a ruptured aneurysm on imaging. Given the improved survival of patients with prostate cancer, radiologists may encounter such unusual cerebral metastases from prostate cancers more frequently in the future.

## Background

Prostate cancer is considered the second most common and fifth most aggressive neoplasm in men globally. It includes 7.1% of all cancers and 3.8% of the related deaths. Overall, one out of seven men is diagnosed with prostate cancer in his lifetime in the USA [1]. Usually, prostate cancer presents with an elevated prostatic specific antigen (PSA) in the blood, with the ultimate diagnosis made via the biopsy. The most common metastatic sites for prostate cancer are the bones (84%), distant lymph nodes (10.6%), liver (10.2%), and lungs. Brain parenchymal metastases from prostate cancer are uncommon because the brain is relatively resistant to them, perhaps due to the blood-brain barrier. The majority of intracranial prostatic metastases involve the dura mater [2]. Brain parenchymal metastases from prostate cancers have been reported in 0.3–3.1% of cases, usually occurring during the disease’s end-stage [3]. Recently, the rate of brain metastases from prostate cancers has risen, likely because of improved survival [4, 5]. Magnetic resonance imaging (MRI) remains the gold standard to evaluate the number, size, and location of brain metastases. Nevertheless, the MRI images of prostatic brain metastases are highly variable and appear from purely solid, to mixed cystic and solid masses, to lesions with rim enhancement.

This report presents a case of cerebral metastasis from prostate cancer with intraventricular bleeding, acute hydrocephalus, and a hemorrhagic exophytic hypothalamic/suprasellar mass.

This uncommon metastasis, mimicking a giant thrombotic aneurysm, deserves to be shared with radiologists through this case report.

## Case presentation

### Patient history

The patient was a 77-year-old man, a known prostate cancer case, who presented to the emergency department with acute onset of severe headache. This patient had a history of chronic hypertension and morbid obesity. Upon the initial examinations, the informed consent form was reviewed and signed by the patient. He was afebrile with a blood pressure reading of 144/79 mmHg, and there was no evidence of altered mental status, sensory or motor deficit noted on admission. Shortly after the initial examinations, the patient became unresponsive and was, therefore, intubated immediately. The lab workup was negative, except for hypernatremia at 154 mmol/L. The COVID-19 screening test was also negative.

### CT imaging

**Fig. 1:**
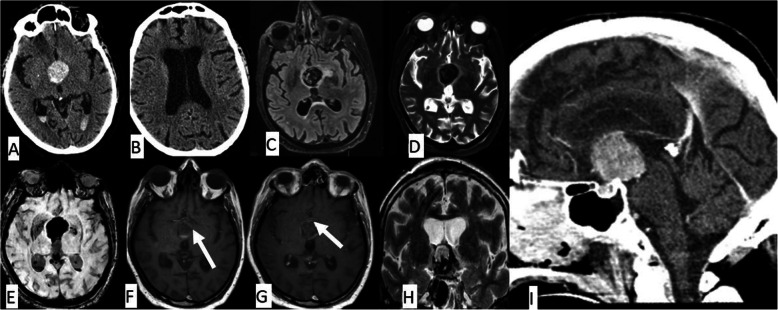
Axial non-contrast CT shows a well-defined hyperdense lesion in the suprasellar cistern associated with intraventricular hemorrhage and acute hydrocephalus (**a**, **b**). The lesion is seen as low signal on FLAIR (**c**) and T2 (**d**) with a signal drop on SWI (**e**). The lesion shows laminar morphology on T1 (**f**) and post-contrast T1 (**g**) but without enhancement. On the coronal T2 (**h**), the lesion appears as an exophytic mass from the hypothalamus with peripheral parenchymal edema. On the sagittal post-contrast CT (**i**), there is a large hyper-dense suprasellar mass with mass effect (compression) over the third ventricle

The patient underwent non-contrast brain CT scanning as the standard workup for thunderclap headache and altered mental status. The CT images revealed a 2.9 × 2.8 cm well-defined, hyper-attenuating lesion, located superior to the basilar tip, anterior to the midbrain, and extending to the suprasellar region without enhancement after the contrast injection (Fig. 1a, b, i).

### Diagnostic assessments

The lesion was interpreted as being a giant thrombosed aneurysm. There was a moderate amount of intraventricular hemorrhage observed with moderate acute hydrocephalus. Also, a peri-ventricular hypo-density was noted, suggestive of sub-ependymal interstitial edema. The subsequent CT angiograms demonstrated patent and normal intracranial arteries without a definitive arterial feeding into the hyper-dense suprasellar lesion. No obvious enhancement within this lesion was noted. The CTA was reported as a completely thrombosed giant aneurysm. The lack of visible vascular communication to the lesion was attributed to the thrombotic aneurysm.

On the subsequent T1 and T2 MR images (Fig. 1c–h), the lesion appeared heterogeneous with foci of hyper-intensities and laminar morphology, compatible with thrombosis. The lesion appeared as a strong signal drop on the sequence of susceptibility-weighted images (SWI). Also, no definitive enhancement was noted after contrast injection, and mild parenchymal edema was noted around the lesion. No definitive vascular communication was noted in the MR images either.

On coronal and sagittal MR images, the lesion was noted to abut the hypothalamus. Upon multidisciplinary workup and the lesion’s proximity to the hypothalamus and the associated edema, it was determined to be a hemorrhagic exophytic hypothalamic mass. Subsequently, the patient underwent ventricular shunting, and a biopsy was taken from the lesion. The pathological findings for the lesion were consistent with a metastatic adenocarcinoma originated from the prostate cancer.

## Discussion

More than 50% of neoplasms in the brain are metastatic, and about 40% of malignancies are complicated with brain metastasis in their course. Brain metastases often happen in end-stage malignancies within 1–2 months of survival without treatment and 6 months of survival with treatment. The most common brain metastases are from the lungs, kidneys, breasts, and melanomas or colorectal neoplasms [6, 7]. Bleeding into intracranial neoplasms happens in 1.3% to 14.6% of intracranial metastases [8]. Hemorrhagic brain parenchymal metastases are well-known hyper-vascular conditions, occurring in melanomas, renal cell carcinomas, choriocarcinomas, and in cancers of breasts and thyroid gland.

Several mechanisms for bleeding within the cerebral metastases have been proposed, including over-expression of vascular endothelial growth factor, imbalances in the fibrinolytic cascade, rapid tumor growth and vascular invasion, neo-vascularization, and tumor necrosis [9]. Despite the high prevalence of prostate cancers, cerebral parenchymal metastases from prostate cancers are uncommon and usually happen in patients with other osseous and soft tissue metastases. In recent years, the increased incidence of brain parenchymal metastases from prostate cancers might be due to improved survival secondary to effective hormonal, radiation, and immune therapies. The most common presentation in these patients is nonfocal neurologic symptoms related to intracranial hypertension. These include confusion, headache, and memory deficits. However, hemiparesis, seizures, and aphasia are less common. The parenchymal metastases are often located in the supratentorial rather than infratentorial area [10].

It has been reported that one third of the prostatic metastases to the brain are hemorrhagic and 19% are cystic or necrotic [11]. Micro-hemorrhages are usually seen on SWI and gradient recalled echo (GRE) sequences. Another intracranial presentation of prostate cancer is metastasis to the dura mater. In this context, prostate cancer is the most common malignancy with dural metastases, followed by breast and lung malignancies [12]. Despite the high prevalence of micro-hemorrhages in prostatic parenchymal metastases [13], subarachnoid and intraventricular hemorrhages are very rare. To our knowledge, only one case of intraventricular hemorrhage has been reported due to prostatic metastasis since the 1980s [13].

### Strength of the study

The current case report is unique in many ways, based on the CT and MR images. These include the initial intraventricular hemorrhage, acute hydrocephalus, and a large exophytic hemorrhagic metastasis from the hypothalamus extended to the suprasellar cistern, with the morphology suggestive of a giant thrombotic aneurysm. In this case, the lesion’s location being adjacent to the hypothalamus, peripheral parenchymal edema, lack of subarachnoid hemorrhage, and lack of visible feeding artery were the helpful information to exclude a possible aneurysm. Lastly, the hypernatremia found in this case was likely due to the patient’s history of diabetes insipidus. To the best of knowledge, this is the first case of exophytic hypothalamic prostatic metastasis that mimicked a ruptured aneurysm.

### Limitation of the study

Unfortunately, the patient passed away after the intraventricular shunt placement and biopsy of the hypothalamic lesion. Therefore, no additional imaging, such as digital subtraction angiography (DSA), is available and the extent of the systemic metastasis remained unknown.

### Recommendation for future research

Radiologists are advised that not every hemorrhagic cerebral lesion with laminar morphology is because of a thrombotic aneurysm, and similar morphology may occur due to intra-axial brain lesions including metastases.

## Conclusions

Brain metastases from prostate cancer may present with intraventricular hemorrhage and acute hydrocephalus. Also, they may mimic giant thrombotic aneurysms because of the clot’s laminar morphology in an exophytic metastasis. In this case, the location and morphology of the metastasis mimicked a giant thrombotic aneurysm, based on the CT and MR images. Given the increasing survival of patients with prostate cancers, radiologists may encounter the unusual presentation of cerebral metastases from these cancers more frequently in the future.

## Data Availability

Supplemental data, including a video file of the brain CT images, are available to the reviewers upon request.

## Abbreviations

COVID-19: Coronavirus disease 2019
CT: Computed tomography
CTA: Computed tomography angiogram
DSA: Digital subtraction angiography
FLAIR: Fluid-attenuated inversion recovery
GRE: Gradient recalled echo
MRI: Magnified resonance imaging
PSA: Prostatic specific antigen
SWI: Susceptibility-weighted images
